# MRANet: Multi‐Dimensional Residual Attentional Network for Precise Polyp Segmentation

**DOI:** 10.1049/syb2.70031

**Published:** 2025-08-17

**Authors:** Li Zhang, Yu Zeng, Yange Sun, Chengyi Zheng, Yan Feng, Huaping Guo

**Affiliations:** ^1^ School of Computer and Information Techonology Xinyang Normal University Xinyang China; ^2^ School of Computer Science and Artificial Intelligence Zhengzhou University Zhengzhou China; ^3^ Information Center Xinyang Agricultural and Forestry University Xinyang China

**Keywords:** feature extraction, image segmentation, medical image processing

## Abstract

Automated polyp detection plays a critical role in the early diagnosis of colorectal cancer, ranking as the second leading cause of cancer‐related mortality worldwide. However, existing segmentation methods face difficulties in handling complex polyp shapes, size variations, and generalising across diverse datasets. We propose a Multi‐dimensional Residual Attention Network (MRANet) for the polyp segmentation task, focusing on enhancing feature representation and ensuring robust performance across diverse clinical scenarios. During encoding, MRANet employs residual self‐attention to capture semantic information of high‐level features, guiding the refinement of low‐level information. In addition, convolutions with Multiple Kernel and Dilation rates (CMKD) are integrated with residual channel and spatial attentions to expand the model's receptive field, enhance encoder features, and accelerate convergence. In the decoding stage, MRANet uses the proposed Attention‐based Scale Interaction Module (ASIM) to merge upsampled high‐level features with low‐level pixel information, enriching low‐level layers using semantic knowledge. A Residual‐based Scale Fusion Module (RSFM) is further designed to merge low‐level features, which preserves high‐frequency details including edges and textures. Experiments demonstrate that MRANet effectively segments polyps with varying sizes, indistinct boundaries, and scattered distributions, achieving the best overall performance. Our code is available at https://github.com/hpguo1982/MRANet.

## Graphical abstract

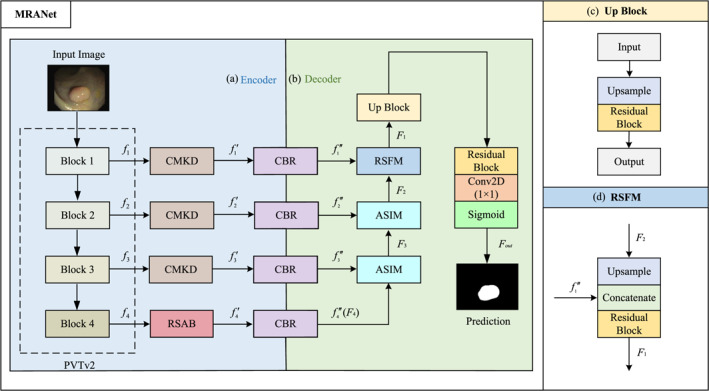

## 1 Introduction

Colorectal cancer (CRC) is a major contributor to cancer‐related mortality globally, posing a substantial burden on public health [1]. Most CRC cases start from polyps in the colon or rectum, which progressively develop into cancer over several years without timely detection. Early detection and removal of these polyps are critical for preventing CRC, highlighting the importance of effective screening methods. Colonoscopy, as the gold standard for colorectal cancer screening, plays a vital role in the detection and diagnosis of polyps, facilitating early intervention and lowering the rates of CRC occurrence and mortality [2]. However, colonoscopy heavily relies on the expertise and experience of the physician. Several factors, such as the complex structure of the colon, potential imaging artefacts, and organ deformation, can increase the risk of missing polyps during the procedure. Therefore, the creation of precise and automated polyp segmentation techniques is essential for early detection and to hinder the advancement of colorectal cancer [3].

Recent advances in deep learning have significantly advanced polyp segmentation tasks, providing effective solutions to mitigate the limitations of conventional colonoscopy [4, 5, 6]. Among these advancements, attention mechanisms and residual structures [7] have emerged as crucial components to facilitate the extraction and integration of enhanced features, allowing the model to capture more relevant information from the backbone network and improve segmentation precision [8, 9].

Attention mechanisms, such as channel‐, spatial‐, and self‐attentions, allow networks to concentrate on critical areas of an image, enhancing their capability to detect and analyse important features. Residual structures, on the other hand, stabilise the network and support the learning of deeper feature representations [10]. Among these mechanisms, the integration of residual structures with channel and spatial attention has been shown to significantly enhance the extraction and integration of features from backbone networks, leading to better performance in polyp segmentation tasks. For example, Tomar et al. [11] introduced an innovative module of the attention mechanism that not only significantly expands the receptive field by applying different proportions of dilation rates during convolution, but also integrates residual connectivity, spatial and channel attention mechanisms. This design allows the model to improve polyp segmentation precision while maintaining computational efficiency. Tomar et al. [12] utilised a pre‐trained ResNet50 to extract features and refine the details of the image through dilated convolutions. Residual connections were employed to integrate features while maintaining network stability. During the decoding phase, channel and spatial attention mechanisms further enhanced feature expression, thereby improving the precision and reliability of polyp segmentation. Jha et al. [13] advanced the learning ability of the network and the transmission of characteristics through residual connections. In addition, they incorporated modules for the attention mechanism, such as the squeeze and excitation network [14] and the dilated spatial pyramid clustering [15], which significantly improves the recognition and processing capabilities of the network for key semantic information.

Many polyp segmentation models effectively combine self‐attention mechanisms with residual structures to leverage their complementary strengths. Specifically, self‐attention mechanisms allow the model to capture long‐range dependencies by emphasising critical regions in the image, whereas residual structures address challenges such as gradient vanishing or explosion by facilitating efficient gradient flow during backpropagation [16]. For example, Fan et al. [17] employed a reverse attention mechanism to extract boundary cues. Residual connections were used to improve feature transfer, establishing a robust link between regions and boundary cues, which improved boundary recognition and segmentation accuracy. By integrating residual and attention mechanisms, their design enables deeper feature learning and more precise image segmentation while maintaining computational efficiency. Similarly, Tomar et al. [18] proposed a feedback attention network for the biomedical image segmentation. The Residual Transformer Block proposed by Jha et al. [19] is a state‐of‐the‐art image analysis module that incorporates the depth perception capability of the self‐attention mechanism with the enhanced feature transfer function of residual networks. This innovative design can accurately capture the complex relationships between points on the feature map and significantly enhance the integration of features through residual connections.

However, traditional segmentation methods still face considerable challenges in handling issues such as blurred polyp boundaries, diverse morphologies, and significant scale variations, particularly when generalising across multi‐source datasets. These challenges not only limit the model's ability to accurately perceive critical regions but also disrupt the balance between preserving local details and capturing global semantic information, thereby reducing the robustness and generalisation capability of the segmentation performance.

Based on the discussion above, a novel Multi‐dimensional Residual Attentional Network (MRANet) is proposed, utilising attention mechanisms and residual connections for precise polyp segmentation. During the encoding process, a Residual Self‐Attention Block (RSAB) is introduced to extract semantic insights from high‐level features of the backbone network, guiding and refining low‐level features. This mechanism enhances the model's ability to optimise the feature extraction process by adaptively adjusting attention weights. In addition, we use Convolutions with Multiple Kernel and Dilation rates (CMKD), followed by the Residual Channel and Spatial Attention (RCSA), to expand the model's receptive field, enhance encoder features, and accelerate model convergence. During the decoding phase, an Attention‐based Scale Interaction Module (ASIM) is designed to fuse features. This innovative module seamlessly integrates upsampled high‐level features with low‐level pixel information, significantly enriching the semantic content of low‐level layers and improving the model's ability to capture fine details. Additionally, we propose a simple Residual‐based Scale Fusion Module (RSFM) to integrate low‐level features, preserving high‐frequency details for finer textures and sharper edges. Leveraging residual connections, RSFM facilitates deeper network, faster convergence, and effective multiscale feature fusion.

In summary, the main contributions of this study are as follows:

- We propose a novel network, called MRANet, for the polyp segmentation task. We integrate a RSAB to harness the global semantic information by calculating the autocorrelation between the high‐level features, and employ a CMKD with RCSA to expand the receptive field, enabling the captures of richer and more reliable features.
- We propose an ASIM to aggregate high‐level features and capture the relationship between different features, and design RSFM for efficient integration of low‐level features, preserving high‐frequency details and ensuring finer textures and sharper edge.
- MRANet achieves superior performance by outperforming nine standard benchmarking methods on an in‐distribution dataset. Evaluations on two out‐of‐distribution datasets highlight MRANet's strong generalisation capabilities. Additionally, heatmap visualisations further reveal that MRANet effectively focuses on the target polyp areas and their edges.

The remainder of the paper is organised as follows: Section 2 describes related works, Section 3 introduces our MRANet, Section 4 presents the experimental results, Section 5 discusses the entire paper, and finally, the work is summarised in Section 6.

## 2 Related Work

### 2.1 Polyp Segmentation

Colorectal cancer (CRC) is a frequent cancer, and timely identification and management of colorectal polyps is able to dramatically reduce the risk of developing CRC [20, 21]. However, accurately segmenting polyps remains a challenging task due to their varying sizes and shapes, along with the unclear boundaries between polyps and the adjacent mucosa [22]. In recent years, image segmentation techniques based on deep learning, especially convolutional neural networks (CNNs), have demonstrated exceptional performance in polyp segmentation benchmarks [23, 24, 25, 26, 27, 28, 29, 30, 31]. Among these methods, U‐Net [32] stands out by leveraging skip connections to aggregate multiscale features, enabling the generation of high‐resolution segmentation results. To further improve segmentation accuracy and efficiency, several U‐Net variants have been developed. For instance, U‐Net++ [27] enhances feature aggregation through nested skip pathways, U‐Net 3+ [33] incorporates deep supervision for better feature fusion, and ResU‐Net++ [34] integrates residual connections to strengthen gradient flow and feature learning. These innovations have led to notable progress in medical image segmentation. Yet, despite the strong performance of CNNs in polyp segmentation, they face inherent limitations in addressing long‐range pixel dependencies limited by the localised scope of convolutions [35]. To address these challenges, researchers have explored advanced networks such as Pyramid Vision Transformer (PVT), which leverages self‐attention mechanisms to capture global context in images [36]. By integrating PVT as a backbone network, models have delivered notable advancement in segmentation precision and generalisation ability, effectively recognising diverse polyp types and addressing the challenges of ambiguous boundaries and variable shapes.

This paper proposes a novel network called MRANet. Like U‐Net and its variants, MRANet emphasises multiscale feature extraction and fusion. Moreover, MRANet introduces Residual Self‐Attention Block (RSAB) to extract global semantic information from high‐level features and employs Convolutions with Multiple Kernel and Dilation rates (CMKD) to expand the receptive field, enabling the capture of richer and more contextualised features. Additionally, Attention‐based Scale Interaction (ASIM) module is employed to aggregate multi‐scale high‐level features, and Residual‐based Scale Fusion Module (RSFM) with residual blocks that incorporate upsampling operations is used to effectively integrate low‐level features, resulting in more accurate and robust segmentation outcomes.

### 2.2 Attention Mechanism

The basic principle of the attention mechanism is to imitate the focusing characteristics of human visual attention, enabling the model to recognise and concentrate on the most critical regions of the image [37]. This mechanism is particularly important in deep learning models because it allows the model to dynamically allocate weights to highlight key features and ignore irrelevant information. In the fields of natural language processing and computer vision, the attention mechanism is usually implemented by calculating the correlation between elements in the input sequence or feature map [38]. For example, in Transformer [39], the self‐attention layer can capture the relationship between any two elements in the sequence, thereby achieving global dependency modelling. In image segmentation tasks, the attention mechanism can help the model identify areas of interest, such as polyps or other lesions in medical images. The key advantage of the attention mechanism is its flexibility and adaptability [40]. It does not rely on fixed rules or preset features, but adjusts dynamically according to the context of the input data. This makes the model better able to handle complex image structures and improves the recognition ability of polyps of different types and sizes [41]. With in‐depth research, the attention mechanism has developed various variants, such as channel attention and spatial attention, which have shown their respective advantages in different application scenarios [42]. By integrating these mechanisms into existing network architectures, the performance of the model can be further enhanced, especially in medical image analysis tasks that require fine segmentation and high precision.

Some researchers have begun to incorporate attention mechanisms into their model architectures to enhance the expressiveness of feature maps, thereby achieving more accurate recognition in pixel‐level classification of medical images [43]. This innovative approach not only captures global information more effectively, significantly improving segmentation performance, but also improves the learning of long‐distance dependencies, providing more precise segmentation results. This has a far‐reaching impact on medical image analysis, promoting the field to advance towards higher levels of accuracy and efficiency. For example, Tomar et al. [44] integrated residual learning, self‐attention mechanisms, and dilated convolution techniques. These approaches were designed to enhance the model's capability to process both local and global features, which plays a critical role in the early identification and treatment of colorectal cancer. Huang et al. [45] adopted the concept of HarDNet68 [46], utilising depthwise separable convolutions and linear bottleneck structures to minimise parameters and decrease computational overhead. They integrated the Receptive Field Block (RFB) module into the skip connections to expand the receptive field of feature maps at different resolutions, thereby enhancing the model's precision in the polyp segmentation task. Jha et al. [47] utilised residual blocks containing Squeeze‐Excitation (SE) blocks to construct the decoder for extracting and merging outputs from the encoder (backbone network). These SE blocks adaptively calculate the importance of each channel within the feature map, thereby enhancing the representational power of the feature map on polyp images. PraNet [17] employed a reverse attention module that extracts boundary information using global feature maps created by high‐level feature decoders.

In this paper, the proposed Multi‐dimensional Residual Attentional Network (MRANet) enhances polyp segmentation by integrating attention mechanisms and residual connections. In particularly, MRANet introduces a Residual Self‐Attention Block (RSAB) to refine high‐level features and guide semantic information extraction. Meanwhile, convolutions with Multiple Kernel and Dilation rates (CMKD) expand the receptive field, improving feature representation. Attention‐based Scale Interaction Module (ASIM) and Residual Scale Fusion Module (RSFM) are used to integrate multiscale features, ensuring precise incorporation of high‐level semantic details.

## 3 Method

### 3.1 Overall Architecture

**FIGURE 1:**
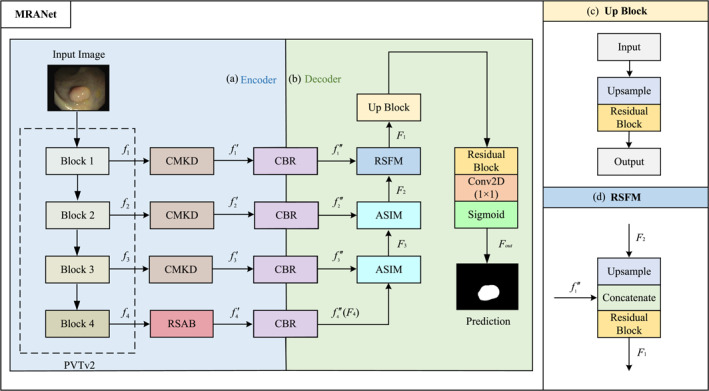
The framework of MRANet. MRANet follows the encoder‐decoder structure, with the encoder filtering low‐level features and high‐level features through CMKD and RSAB modules, respectively, and with the decoder fusing the filtered high‐level features and low‐level features through ASIM and RSFM modules. CBR, Convolution + Batch normalisation + ReLU activation.

The architecture of our MRANet is illustrated in Figure 1. MRANet employs PVTv2 [36], pretrained on ImageNet, as the backbone for its efficient extraction of rich multiscale features, ensuring a robust foundation for downstream tasks. The outputs of the four fundamental blocks at the side end of the backbone are denoted as $f_{i}$, where i∈{1,2,3,4} represents the index of each block. To further enhance the representational power of the former three features, that is, $f_{1},f_{2}$, and $f_{3}$, CMKD are introduced to capture intricate local details and improve the model's sensitivity to subtle image features, generating the processed features $f_{i}^{\prime },i=1,2,3$. Additionally, a RSAB is utilised on the top‐level feature $f_{4}$ to obtain $f_{4}^{\prime }$, allowing global contextual modelling while preserving critical high‐level semantic information. Then, each $f_{i}^{\prime }$ goes through a Convolution with Batch normalisation and ReLU activation (CBR), generating the refined feature $f_{i}^{\prime }$. Subsequently, the refined features are fed into the designed decoder structure. We propose ASIM to facilitate the efficient fusion of multiscale high‐level features. During the decoding process, ASIM plays a crucial role in recovering fine details, particularly in medical images with complex textures and boundaries, thus improving detail clarity and improving segmentation accuracy. In addition, we develop a RSFM to merge low‐level features, retaining high‐frequency components to enhance texture richness and edge sharpness. By incorporating residual connections, RSFM facilitates deeper network designs, accelerates training, and ensures efficient multiscale feature integration.

### 3.2 Encoder Network

The encoder network, as shown in Figure 1a, employs PVTv2 as the backbone for feature extraction. We use a RSAB to mine the global semantic information by calculating the autocorrelation of the top‐level feature, and employ a Convolutions with CMKD to broaden the receptive field and capture richer and more reliable characteristic of low‐level features. Formally, let $x\in R^{c\times h\times w}$ represent an input image with c channels, height h, and width w. We denote $f_{i}$ as the output of *i*th Block, i=1,2,3,4. Then, the outputs of the encoder is expressed as follows:

(1) $$f_{i}^{\prime }=\left\{\begin{array}{cc} \text{RSAB}\left(f_{i}\right),\; & \text{if}\;i=4,\\ \text{CMKD}\left(f_{i}\right),\; & \text{else} \end{array}\right.$$

#### 3.2.1 Residual Self‐Attention Block

**FIGURE 2:**
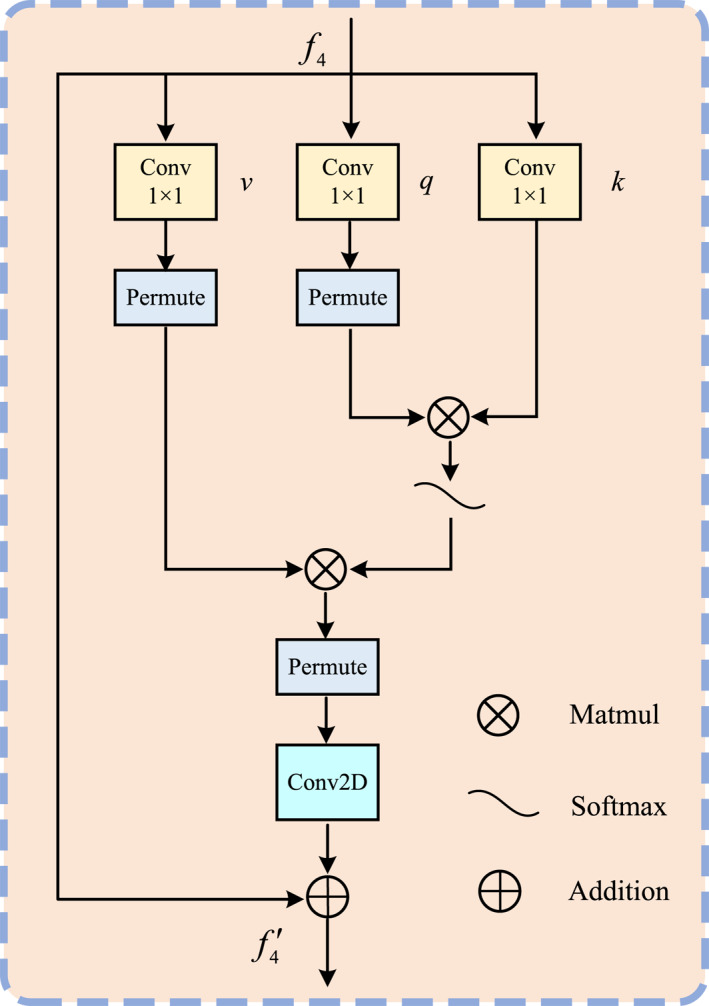
The structure of RSAB.

In this section, we employ self‐attention to extract semantic cues from high‐level features, facilitating the refinement of low‐level information. Figure 2 shows the designed RSAB. During the initialisation phase, essential parameters are configured, including the number of channels, padding, and grouping, and dimensionality reduction techniques are employed to minimise computational overhead. This process primarily involves the generation of the query, key, and value tensors via 1×1 convolution operations described as follows:

(2) $$\begin{array}{ccc} v & = & Reshape\left(Conv_{1\times 1}\left(f_{4}\right)\right)\\ q & = & {\left(Reshape\left(Conv_{1\times 1}\left(f_{4}\right)\right)\right)}^{T}\\ k & = & Conv_{1\times 1}\left(f_{4}\right) \end{array}$$

Here, T denotes matrix transposition. Attention weights are derived through matrix multiplication and a subsequent softmax operation. These weights adjust the output features, which are then reshaped, processed with convolution, and combined with $f_{4}$ to yield the final result. Formally, we have

(3) $$f_{4}^{\prime }=f_{4}+\text{Conv}\left(\text{Reshape}\left(v⊗\text{Softmax}\left(q⊗k\right)\right)\right)$$

#### 3.2.2 Convolutions With Multiple Kernels and Dilation Rates

**FIGURE 3:**
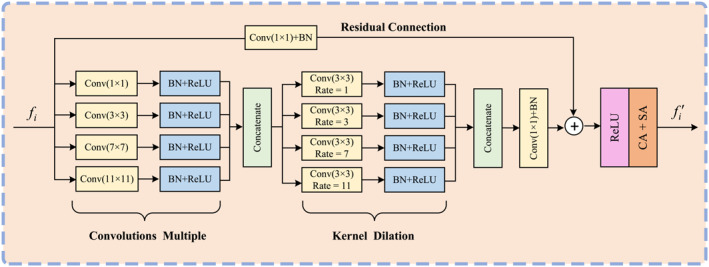
The structure of CMKD.

Although the self‐attention mechanism improves the model's performance, it ignores the fact that spatial locality is the main characteristic of the image and that effectively capturing the localisation characteristic is essential for the success of computer vision tasks. To address this limitation, we propose the convolutions with CMKD module, illustrated in Figure 3.

The CMKD module strengthens the network's ability to capture fine details by extracting multiscale features in parallel using convolutional kernels of varying sizes, ranging from 1×1 to 11×11. Each convolution is paired with batch normalisation (BN) and ReLU activation, facilitating faster training and improved stability. Let $f_{i}\in R^{H\times W\times C}$ represent the input feature, the result of the j‐th convolution is expressed as follows:

(4) $$f_{i,j}=ReLU\left(BN\left({Conv}_{k_{j}\times k_{j}}\left(f_{i}\right)\right)\right)$$

Here, $k_{j}\times k_{j}$ denotes the kernel size for the j‐th convolution, with $k_{1}=1$, $k_{2}=3$, $k_{3}=7$, and $k_{4}=11$. The resulting features are concatenated to form:

(5) $$f_{\text{con}}=Concat\left(f_{i,1},f_{i,2},f_{i,3},f_{i,4}\right)$$

The concatenated feature $f_{\text{con}}$ is refined using four parallel dilated convolution operations with dilation rates of $r_{1}=1$, $r_{2}=3$, $r_{3}=7$, and $r_{4}=11$. The output of each dilated convolution operation is defined as follows:

(6) $$f_{i,j}^{\prime }=ReLU\left(BN\left({Conv2D}_{3\times 3}\left(f_{\text{con}},r_{j}\right)\right)\right)$$

Subsequently, a 1×1 convolution integrates these multiscale features, allowing the network to learn deeper representations:

(7) $$f_{\text{main}}={Conv}_{1\times 1}\left(Concat\left(f_{i,1}^{\prime },f_{i,2}^{\prime },f_{i,3}^{\prime },f_{i,4}^{\prime }\right)\right)$$

CMKD incorporates residual connections to combat gradient vanishing in deeper networks, enabling the network to preserves shallow feature significance while enhancing deeper feature representations. Formally,

(8) $$f_{\text{main}}^{\prime }=ReLU\left(f_{\text{main}}+BN\left({Conv}_{1\times 1}\left(f_{i}\right)\right)\right)$$

Finally, channel and spatial attention mechanisms [48] are employed to refine $f_{\text{main}}^{\prime }$, highlighting important channels and spatial regions to improve the robustness and precision of the features:

(9) $$f_{i}^{\prime }=f_{\text{main}}^{\prime }+SA\left(CA\left(f_{\text{main}}^{\prime }\right)\right)$$

### 3.3 Decoder Network

**FIGURE 4:**
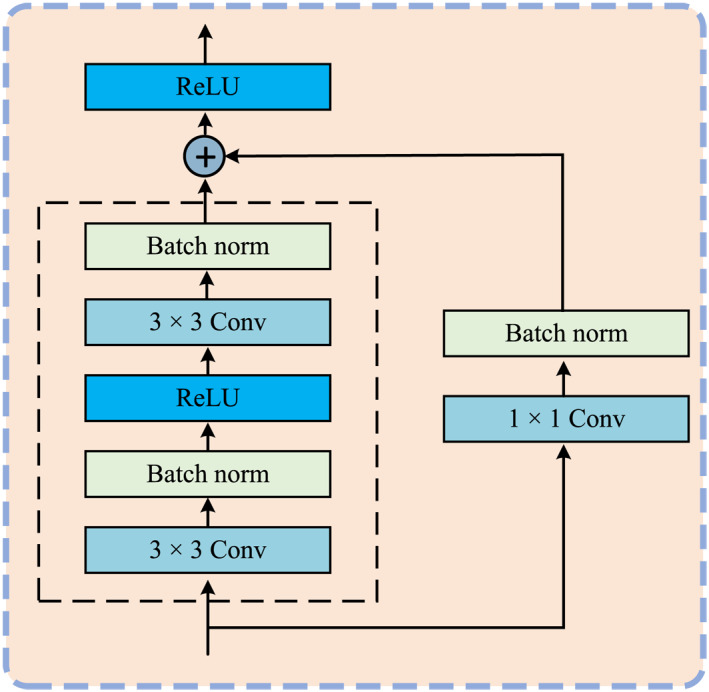
The structure of a residual block.

The constrained receptive field of convolution operations makes it difficult to capture global context, which is essential for comprehensive feature representation. To address this problem, convolutional networks leverage the complementary nature of features across different layers: high‐level layers predominantly encode semantic information, while low‐level layers retain detailed spatial structures. According to this observation, we design an ASIM to integrate high‐level interlayer features effectively. Furthermore, a RSFM is employed to fuse low‐level features, enabling the progressive reconstruction of saliency information. Finally, a general upsampling block, combined with residual structure, is introduced to enhance the precision of saliency map details. Using the complementarity of high‐ and low‐level features, the proposed method achieves robust and accurate lesions detection. Formally,

(10) $$F_{i}=\left\{\begin{array}{cc} f_{i}^{\prime \prime },\; & \text{if}\;i=4,\\ \text{ASIM}\left(f_{i}^{\prime \prime },F_{i+1}\right),\; & \text{if}\;i=2,3\\ \text{RSFM}\left(f_{i}^{\prime \prime },F_{i+1}\right),\; & \text{if}\;i=1 \end{array}\right.$$

(11) $$F_{\text{out}}=\sigma \left({\text{Conv}}_{1\times 1}\left(\text{RB}\left(\text{Up}\left(F_{1}\right)\right)\right)\right)$$

where $f_{i}^{\prime \prime }$ is the input to the decoder's *i*th layer, as discussed in Section 3.1, RB is the residual structure (Figure 4), and Up is the operations of the upsampling block (Figure 1c).

#### 3.3.1 Attention‐Based Scale Interaction Module

**FIGURE 5:**
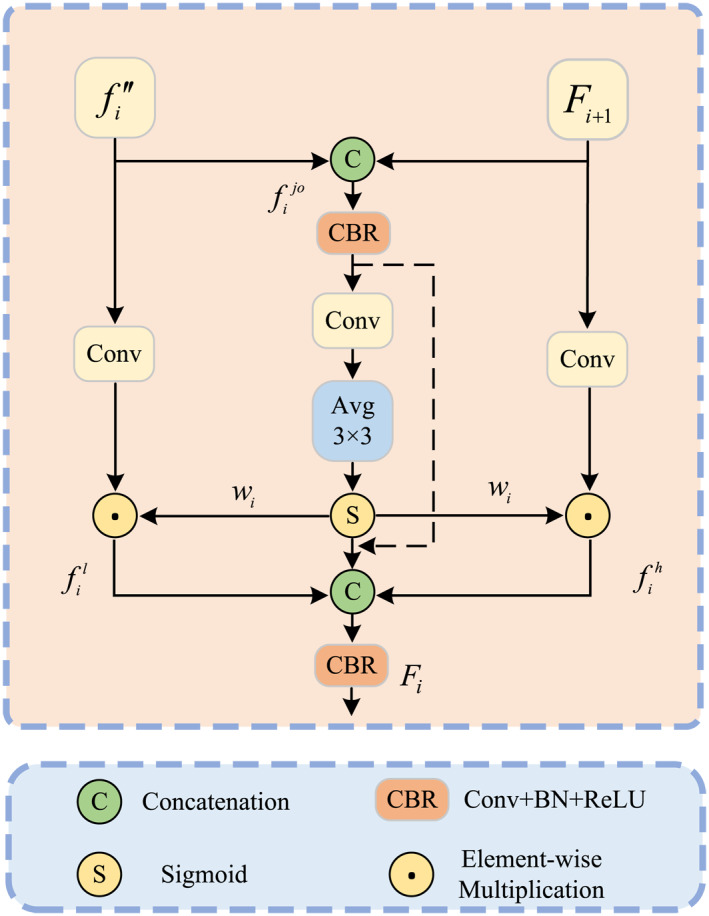
The structure of ASIM.

As shown in Figure 5, the proposed ASIM enhances feature extraction by fusing feature from different layers. The inputs of the ASIM module are high‐level feature $F_{i+1}$ and low‐level feature $f_{i}^{\prime \prime }$ [refer to Section 3.1 and Figure 1b]. The inputs are first unified to the same resolution and merged at the join point to generate the initial fusion feature $f_{i}^{jo}$:

(12) $$f_{i}^{jo}=\text{Concate}\left(\text{Upsample}\left(F_{i+1}\right),f_{i}^{\prime }\right)$$

where $f_{i}^{jo}$ is processed through a CBR block, followed by a convolution and average pooling for dimensionality reduction. Then we obtain a weight graph $w_{i}$ with rich information as follows:

(13) $$w_{i}=\sigma (\text{Avg}(\text{Conv}(\text{CBR}(f_{i}^{jo}))))$$

We use $w_{i}$ to weight the low‐features $f_{i}^{\prime }$ and the high‐level features $F_{i+1}$, and fuse them to obtain a feature $f_{\mathrm{out}}$:

(14) $$f_{i}^{l}=w_{i}⊙\text{Conv}\left(f_{i}\right)$$

(15) $$f_{i}^{h}=w_{i}⊙\text{Conv}\left(F_{i+1}\right)$$

(16) $$F_{i}=\text{CBR}(\text{Concate}(f_{i}^{l},f_{i}^{h},f_{i}^{jo}))$$

By combining these features, that is, $f_{i}$ and $F_{i+1}$, within the ASIM, their complementarity can be fully utilised, allowing simultaneous representation of detailed structures and global semantics. This fusion significantly improves the quality of the feature representation, providing stronger support for subsequent tasks such as saliency reconstruction and lesions location with greater precision.

#### 3.3.2 Residual‐Based Scale Fusion Module

As shown in Figure 1d, the RSFM combines upsampling and residual blocks to augment the resolution and feature representation.

First, the input feature $F_{2}$ is enlarged using a bilinear upsampling layer:

(17) $$F_{2}^{\text{up}}=\text{Upsample}\left(F_{2}\right)$$

$F_{2}^{\text{up}}$ is then concatenated with $f_{1}^{\prime \prime }$ to form new feature $f_{1}^{\text{con}}$:

(18) $$f_{1}^{\text{con}}=\text{Concate}\left(F_{2}^{\text{up}},f_{1}^{\prime \prime }\right)$$

Next, $f_{1}^{\text{con}}$ is directly passed into a residual block for further processing, expressed as follows:

(19) $$F_{1}=RB\left(f_{1}^{\text{con}}\right)$$

where RB(⋅) represents the residual block processing, as shown in Figure 4. The residual block solves the gradient issue during deep network learning by adding shortcut paths to maintain learning stability. A standard residual block is composed of consecutive convolution, batch normalisation, and ReLU function with dimensionality adjusted by 1×1 convolution for shortcut connections. This design simplifies the optimisation process and allows the network to learn the residual mapping without additional parameters, thus improving performance.

### 3.4 Loss Function

An effective loss function can enhance network performance without altering the model architecture. To produce high‐quality segmentation results, we use a hybrid loss for model training, which is formulated as follows:

(20) $$L_{\mathrm{total}}=L_{\text{BCE}}+L_{\text{Dice}}$$

where $L_{\text{BCE}}$ and $L_{\text{Dice}}$ represent the binary cross‐entropy loss [49] and the Dice loss [50], respectively.

## 4 Experiments

### 4.1 Experimental Setup

#### 4.1.1 Datasets

We use multiple publicly available datasets to evaluate the performance of our MRANet on the polyp segmentation task, namely Kvasir‐SEG [51], PolypGen [52], and CVC‐ClinicDB [53].

The Kvasir‐SEG dataset is a medical image segmentation benchmark consisting of real gastrointestinal endoscopy images of various common lesions like polyps and ulcers, with an official data split of 880 training, 120 validation, and 196 test images. The PolypGen dataset comprises a comprehensive collection of endoscopic images provided by multiple medical institutions. The dataset covers a diverse range of populations, device types, and acquisition conditions. Six subsets are included in the dataset, labelled C1 through C6. The CVC‐ClinicDB dataset is a medical imaging dataset specifically designed for colorectal cancer detection research, comprising 612 high‐resolution colonoscopy images.

#### 4.1.2 Implementation Details

Our experiments were conducted based on the PyTorch framework and carried out on a system equipped with an NVIDIA A100 GPU. The Adam optimiser is employed with a learning rate of 1e‐4, a batch size of 16, and a maximum of 150 training epochs. Additionally, to enhance the model's generalisation and increase the diversity of the training dataset, we employ the following data augmentation techniques: random rotation, horizontal flipping, and vertical flipping of images. All comparative and ablation experiments are conducted using the above configuration.

#### 4.1.3 Evaluation Metrics

To thoroughly assess the effectiveness of our MRANet, we adopt an integrated and rigorous assessment framework that incorporates metrics for segmentation accuracy, classification balance, and boundary localisation.

For segmentation accuracy, we use the mean intersection over union (mIoU) [54], defined as follows:

(21) $$\text{mIoU}=\frac{1}{N}\sum_{i=1}^{N}\frac{A_{i}\cap B_{i}}{A_{i}\cup B_{i}},$$

and the mean Dice similarity coefficient (mDSC) [55], expressed as follows:

(22) $$\text{mDSC}=\frac{1}{N}\sum_{i=1}^{N}\frac{2|A_{i}\cap B_{i}|}{\left|A_{i}|+|B_{i}\right|}$$

To balance sensitivity and specificity, we incorporates the Recall and Precision metrics:

(23) $$\text{Recall}=\frac{\text{TP}}{\text{TP}+\text{FN}},\;\text{Precision}=\frac{\text{TP}}{\text{TP}+\text{FP}}$$

Furthermore, the F2‐score, which reconciles Recall and Precision, is included as a comprehensive measure:

(24) $$F_{\beta }=\left(1+\beta^{2}\right)\cdot \frac{\text{Precision}\cdot \text{Recall}}{\beta^{2}\cdot \text{Precision}+\text{Recall}}$$

To evaluate boundary localisation Precision, the framework employs the Hausdorff Distance (HD), allowing for a multidimensional comparison of advanced methods. HD is defined as follows:

(25) $$\text{HD}\left(A,B\right)=\max\left\{\sup_{a\in A}\inf_{b\in B}\|a-b\|,\sup_{b\in B}\inf_{a\in A}\|a-b\|\right\}$$

Here, A and B denote the sets of boundary points from the prediction and the ground truth, respectively. The Hausdorff Distance measures the greatest of all the shortest distances from one set to the other. That is, for each point in A, the distance to the nearest point in B is computed, and vice versa; the maximum of these values is taken as the HD. A smaller HD indicates that the predicted boundary closely aligns with the ground truth, reflecting higher localisation accuracy.

This comprehensive evaluation framework ensures a robust assessment of the model's performance by addressing segmentation accuracy, balancing classification metrics, and precisely localising boundaries.

### 4.2 In‐Distribution Testing

We conduct an in‐distribution performance evaluation of our MRANet using the Kvasir‐SEG dataset. As shown in Table 1, MRANet achieves the best performance with mDSC (0.9479), mIoU (0.9068), Recall (0.9505), Precision (0.9517), and F2‐score (0.9490) on the Kvasir‐SEG dataset. Compared to advanced methods such as TransNetR, G‐CASCADE, and FANetv2, MRANet shows significant advantages across all evaluation metrics, achieving higher segmentation accuracy and greater robustness.

**TABLE 1:** In‐distribution performance evaluation of compared methods on the Kvasir‐SEG dataset.

Method	Backbone	mIoU	mDSC	Recall	Prec.	F2	HD
U‐Net [32]	—	0.7472	0.8264	0.8504	0.8703	0.8353	4.8052
U‐Net++ [27]	—	0.7420	0.8228	0.8437	0.8607	0.8295	4.6904
U‐Net 3+ [33]	—	0.7929	0.8587	0.8518	0.9308	0.8525	4.4677
ResU‐Net++ [34]	—	0.5341	0.6453	0.6964	0.7080	0.6576	4.3089
HarDNet‐MSEG [45]	HardNet68	0.7459	0.8260	0.8485	0.8652	0.8358	4.2036
ColonSegNet [47]	—	0.6980	0.7920	0.8193	0.8432	0.7999	3.9678
TransResU‐Net [44]	ResNet50	0.8214	0.8884	0.9106	0.9022	0.8971	4.8971
TransNetR [19]	ResNet50	0.8016	0.8706	0.8843	0.9073	0.8744	3.9044
G‐CASCADE [56]	PVT	0.8732	0.9201	0.9203	0.9508	0.9154	3.3967
FANetv2 [57]	PVT	0.8704	0.9185	0.9201	0.9405	0.9137	3.3059
**Ours**	PVT	**0.9068**	**0.9479**	**0.9505**	**0.9517**	**0.9490**	**3.2659**

*Note:* Bold values indicate that the corresponding model achieves the best performance on the corresponding metrics.

**FIGURE 6:**
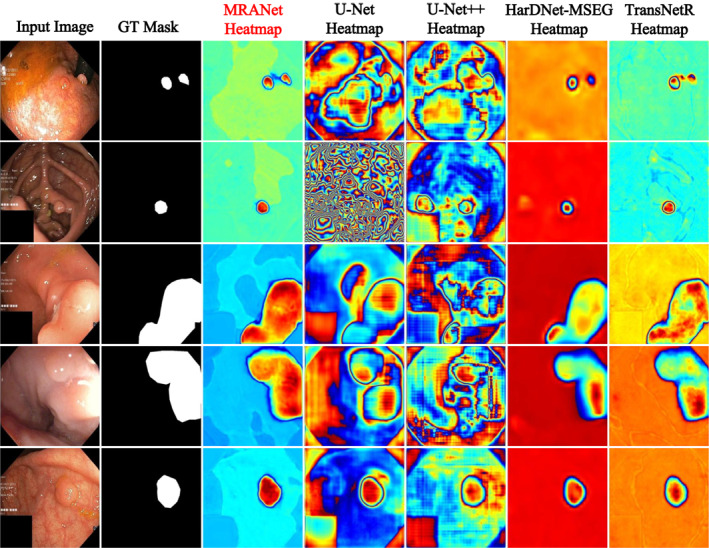
The heatmap represents the density or magnitude of the data through changes in colour.

The Heatmap is an effective way to visualise the distribution of the strength of the model predictions. In medical image analysis, particularly for polyp detection and segmentation, the application of heatmaps is especially important. Figure 6 shows the heatmaps generated by comparing the methods on five polyp images from the Kvasir‐SEG dataset. From Figure 6, the heatmaps produced by our MRANet not only precisely identified the polyp region, but also excelled in boundary details. These details are reflected not only in the overall recognition of the polyp but also extended to the precise capture of the polyp's edge. Compared with other models, the MRANet heatmap more closely matches the actual distribution of the polyp, demonstrating its superior performance on the polyp segmentation task.

**FIGURE 7:**
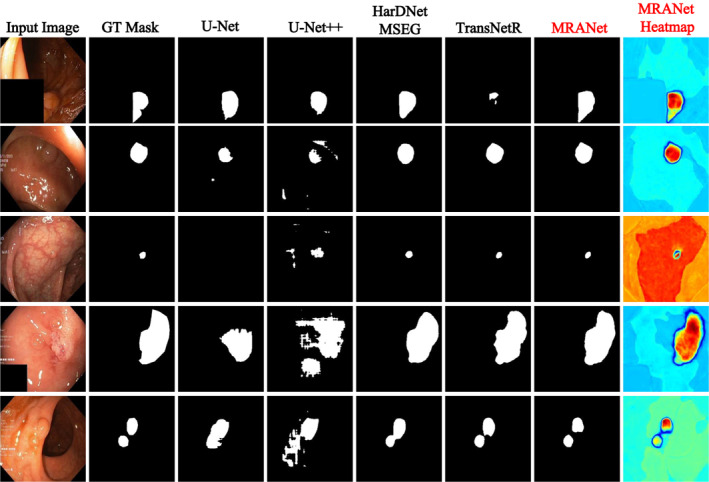
A qualitative example of Kvasir‐SEG polyp segmentation.

The comparison of the segmentation results on five polyp images of the Kvasir‐SEG dataset, as shown in Figure 7, enables us to directly assess the performance of our MRANet. MRANet demonstrates its superior capability in boundary capturing and detail recognition, especially for small polyps that are often overlooked by other segmentation methods. This capability is crucial for the timely detection of cancer, as small polyps may indicate the early stages of the disease.

### 4.3 Out‐of‐Distributuion Testing

**FIGURE 8:**
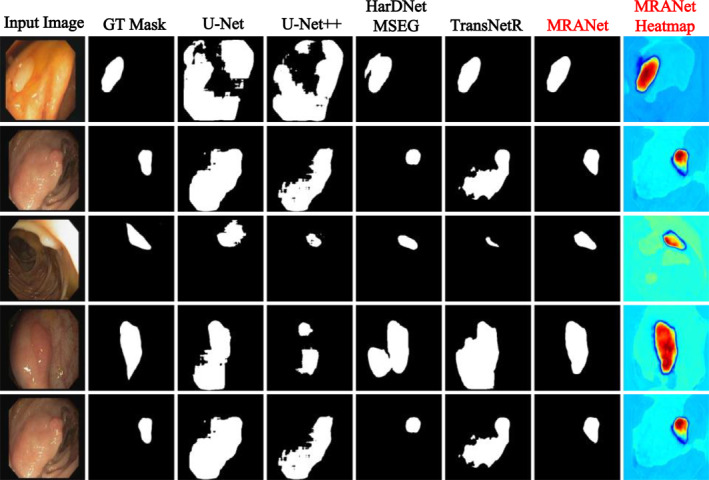
A qualitative example of CVC‐ClinicDB polyp segmentation.

We conduct out‐of‐distribution evaluations of MRANet using both the CVC‐ClinicDB and PolypGen datasets. Table 2 and Figure 8 display the model's overall performance and the visualised segmentation effects on the CVC‐ClinicDB dataset, while Table 3 shows the performance on the PolypGen dataset.

**TABLE 2:** The model is trained on the Kvasir‐SEG dataset and tested on the out‐of‐distribution dataset CVC‐ClinicDB.

Method	Backbone	mIoU	mDSC	Recall	Prec.	F2	HD
U‐Net [32]	—	0.5433	0.6336	0.6982	0.7891	0.6563	5.0396
U‐Net++ [27]	—	0.5475	0.6530	0.6933	0.7967	0.6556	4.9969
U‐Net 3+ [33]	—	0.5196	0.6039	0.7051	0.7336	0.6382	4.6382
ResU‐Net++ [34]	—	0.3585	0.4642	0.5880	0.5770	0.5084	4.8969
HarDNet‐MSEG [45]	HardNet68	0.6058	0.6960	0.7173	0.8528	0.7010	4.7856
ColonSegNet [47]	—	0.5090	0.6126	0.6564	0.7521	0.6246	4.8697
TransResU‐Net [44]	ResNet50	0.6238	0.7011	0.7794	0.7390	0.7380	4.7380
TransNetR [19]	ResNet50	0.6912	0.7655	0.7571	**0.9200**	0.7565	3.9987
G‐CASCADE [56]	PVT	0.7024	0.7761	0.7893	0.8506	0.7846	3.6877
FANetv2 [57]	PVT	0.7103	0.7749	0.7874	0.8637	0.7901	3.7139
**Ours**	PVT	**0.7158**	**0.7889**	**0.7994**	0.8421	**0.7928**	**3.5695**

*Note:* Bold values indicate that the corresponding model achieves the best performance on corresponding metrics.

**TABLE 3:** The model is trained on the Kvasir‐SEG dataset and tested on the out‐of‐distribution dataset PolypGen (C1‐C6).

Method	Backbone	mIoU	mDSC	Recall	Precision	F2	HD
C1							
U‐Net [32]	—	0.5772	0.6469	0.6780	0.8464	0.6484	4.8983
U‐Net++ [27]	—	0.5857	0.6611	0.6953	0.8247	0.6700	4.7442
U‐Net 3+ [33]	—	0.5979	0.6631	0.6960	0.8480	0.6696	4.5920
ResU‐Net++ [34]	—	0.4204	0.5239	0.6390	0.5789	0.5557	5.4847
HarDNet‐MSEG [45]	HardNet68	0.6256	0.7121	0.7800	0.7933	0.7344	4.1428
ColonSegNet [47]	—	0.5514	0.6386	0.7130	0.7423	0.6551	4.9940
TransResU‐Ne	ResNet50	0.7000	0.7708	0.8137	0.8542	0.7854	4.1395
TransNetR [19]	ResNet50	0.6538	0.7204	0.7438	**0.8778**	0.7269	4.1922
G‐CASCADE [56]	PVT	0.7237	0.7864	0.7969	0.8706	0.7969	4.2987
FANetv2 [57]	PVT	0.7389	0.7969	0.8063	0.8891	0.8159	4.3747
**Ours**	PVT	**0.7647**	**0.8374**	**0.8898**	0.8479	**0.8567**	**3.8533**
C2							
U‐Net [32]	—	0.5772	0.6338	0.7347	0.7368	0.6495	4.1535
U‐Net++ [27]	—	0.5612	0.6204	0.7189	0.7631	0.6383	4.3425
U‐Net 3+ [33]	—	0.5844	0.6416	0.6970	0.7986	0.6437	4.0609
ResU‐Net++ [34]	—	0.2779	0.3431	0.5003	0.4189	0.3606	5.2346
HarDNet‐MSEG [45]	HardNet68	0.5667	0.6311	0.7267	0.7149	0.6376	3.4968
ColonSegNet [47]	—	0.4659	0.5371	0.6443	0.6789	0.5439	4.5876
TransResU‐Net [44]	ResNet50	0.5944	0.6594	0.8491	0.6718	0.6971	3.7237
TransNetR [19]	ResNet50	0.6608	0.7203	0.8071	**0.8089**	0.7366	3.5676
G‐CASCADE [56]	PVT	0.7084	0.7379	0.8271	0.7863	0.7412	3.9829
FANetv2 [57]	PVT	0.7194	0.7469	0.8431	0.7764	0.7539	3.7919
**Ours**	PVT	**0.7280**	**0.7860**	**0.8908**	0.8047	**0.7991**	**3.2965**
C3							
U‐Net [32]	—	0.6769	0.7481	0.7637	0.8787	0.7518	4.8423
U‐Net++ [27]	—	0.6530	0.7254	0.7526	0.8568	0.7332	4.7139
U‐Net 3+ [33]	—	0.6739	0.7456	0.7629	0.8703	0.7492	4.1634
ResU‐Net++ [34]	—	0.4096	0.5109	0.6463	0.5484	0.5545	5.3988
HarDNet‐MSEG [45]	HardNet68	0.6623	0.7440	0.7947	0.8180	0.7619	3.6581
ColonSegNet [47]	—	0.6181	0.7064	0.7520	0.7907	0.7221	4.6104
TransResU‐Net [44]	ResNet50	0.7516	0.8247	0.8515	0.8809	0.8346	3.7343
TransNetR [19]	ResNet50	0.7217	0.7874	0.7904	**0.9133**	0.7863	3.7747
G‐CASCADE [56]	PVT	0.7469	0.8314	0.8243	0.8744	0.8326	3.7127
FANetv2 [57]	PVT	0.7619	0.8561	0.8381	0.8814	0.8470	3.6217
**Ours**	PVT	**0.8090**	**0.8755**	**0.9013**	0.8862	**0.8847**	**3.4856**
C4							
U‐Net [32]	—	0.3699	0.4147	0.6550	0.5982	0.4263	3.1962
U‐Net++ [27]	—	0.3807	0.4202	0.6337	0.6099	0.4294	3.0279
U‐Net 3+ [33]	—	0.3909	0.4315	0.6291	0.6592	0.4386	3.0768
ResU‐Net++ [34]	—	0.1689	0.2268	0.6342	0.2816	0.2433	3.6532
HarDNet‐MSEG [45]	HardNet68	0.3516	0.3936	0.6758	0.5535	0.4062	2.7778
ColonSegNet [47]	—	0.2933	0.3244	0.6493	0.4710	0.3558	3.1976
TransResU‐Net [44]	ResNet50	0.4180	0.4690	0.7823	0.5472	0.4937	2.7403
TransNetR [19]	ResNet50	**0.4601**	0.5042	0.6874	**0.7141**	0.5096	2.9209
G‐CASCADE [56]	PVT	0.4389	0.4877	0.7325	0.6137	0.5024	2.8693
FANetv2 [57]	PVT	0.4419	0.4931	0.7543	0.6314	0.5129	2.9413
**Ours**	PVT	0.4514	**0.5057**	**0.8531**	0.5229	**0.5338**	**2.4763**
C5							
U‐Net [32]	—	0.2963	0.3614	0.4577	0.5497	0.3870	4.8963
U‐Net++ [27]	—	0.3143	0.3773	0.4475	0.6030	0.3935	4.6263
U‐Net 3+ [33]	—	0.3216	0.3823	0.4423	0.5927	0.3949	4.7336
ResU‐Net++ [34]	—	0.2041	0.2748	0.4643	0.3027	0.3156	5.3125
HarDNet‐MSEG [45]	HardNet68	0.3090	0.3769	0.4588	0.5250	0.3970	4.4298
ColonSegNet [47]	—	0.2687	0.3416	0.4097	0.5232	0.3532	4.8444
TransResU‐Net [44]	ResNet50	0.3352	0.4014	0.4913	0.5462	0.4213	4.7321
TransNetR [19]	ResNet50	0.3597	0.4214	0.4508	**0.7767**	0.4232	4.4563
G‐CASCADE [56]	PVT	0.4127	0.5216	0.5784	0.7281	0.5328	4.3410
FANetv2 [57]	PVT	0.4328	0.5431	0.5817	0.7372	0.5461	4.6421
**Ours**	PVT	**0.5062**	**0.5866**	**0.6361**	0.7390	**0.5982**	**4.0672**
C6							
U‐Net [32]	—	0.5384	0.6126	0.7054	0.7508	0.6362	4.3135
U‐Net++ [27]	—	0.5355	0.6163	0.7340	0.7230	0.6564	4.2843
U‐Net 3+ [33]	—	0.5387	0.6065	0.7076	0.7297	0.6351	4.0034
ResU‐Net++ [34]	—	0.2816	0.3684	0.6220	0.3526	0.4326	4.9259
HarDNet‐MSEG [45]	HardNet68	0.5548	0.6341	0.7197	0.7722	0.6487	3.4179
ColonSegNet [47]	—	0.4410	0.5290	0.6199	0.6403	0.5424	4.5184
TransResU‐Net [44]	ResNet50	0.6501	0.7151	0.7822	0.8091	0.7331	3.5544
TransNetR [19]	ResNet50	0.6335	0.6917	0.6783	**0.9431**	0.6803	3.6173
G‐CASCADE [56]	PVT	0.6897	0.7236	0.7632	0.7746	0.7458	3.5617
FANetv2 [57]	PVT	0.6991	0.7485	0.7831	0.7917	0.7657	3.4621
**Ours**	PVT	**0.7328**	**0.7941**	**0.8339**	0.8617	**0.8023**	**3.2566**

*Note:* The bold values indicate that the corresponding metric achieves the best performance among all comparison methods.

#### 4.3.1 CVC‐ClinicDB Dataset

As shown in Table 2, MRANet achieves the best performance with mIoU (0.7158), mDSC (0.7889), Recall (0.7994), Precision (0.8421), and F2‐score (0.7928) on the CVC‐ClinicDB dataset. Our MRANet significantly outperforms FANetv2, the closest competitor, in all assessments, with the exception that MRANet is slightly inferior to TransNetR in terms of Precision.

The heatmaps presented in Figure 8 provide an intuitive understanding of MRANet's performance on the CVC‐ClinicDB dataset. In complex images containing multiple polyps, MRANet demonstrated exceptional segmentation capabilities, accurately identifying the location of each polyp and distinguishing them in detail. We observe from Figure 8 that, compared to TransNetR, MRANet shows a significant advantage in boundary recognition, being able to accurately capture the subtle changes that are extremely important in medical image analysis.

#### 4.3.2 PolypGen Dataset

As shown in Table 3, MRANet significantly outperforms the other methods in terms of mIoU, mDSC, Recall, F2‐score, and HD, and is slightly outperformed by TransNetR in Precision. In addition, the generalisation abilities of the eight comparing methods are challenged on PolypGen (C4). Overall, compared to other methods, MRANet demonstrates superior robustness and generalisation ability on the polyp segmentation task.

In summary, MRANet exhibits excellent performance on the in‐distribution dataset Kvasir‐SEG and demonstrated high generalisation ability on out‐of‐distribution datasets CVC‐ClinicDB and PolypGen, proving its potential and reliability in practical applications.

### 4.4 Running Efficiency

To comprehensively evaluate the training efficiency and model complexity of MRANet, we compare it with several mainstream medical image segmentation models. Comparison metrics include the number of training epochs, training time per epoch, total training time, and the number of model parameters. From Table 4, the results demonstrate that MRANet achieves an excellent balance between model complexity and performance, while exhibiting high training efficiency. With 52.38 M parameters, MRANet has fewer parameters than TransNetR, G‐CASCADE, and FANetv2. This indicates that MRANet effectively controls model complexity and offers strong potential for practical deployment.

**TABLE 4:** Comparison of time and the number of parameters for different methods.

Method	Epoch	Training time per epoch(s)	Training time (min)	Total number of parameters (M)
U‐Net [32]	102	25	43	38.42
U‐Net++ [27]	113	28	53	34.15
U‐Net 3+ [33]	99	29	48	42.36
ResU‐Net++ [34]	93	56	87	55.47
HarDNet‐MSEG [45]	94	60	94	58.31
ColonSegNet [47]	89	51	76	53.96
TransResU‐Net [44]	94	58	91	56.28
TransNetR [19]	92	52	80	54.76
G‐CASCADE [56]	91	54	81	57.36
FANetv2 [57]	94	56	87	58.62
**Ours**	88	48	70	52.38

### 4.5 Ablation Study

We show in Table 5 an ablation study to evaluate the impact of different components on the MRANet model, where details of RSAB,CMKD, ASIM, and RSFM are introduced in Section 3. We observe from Table 5 that MRANet performs the best in all the metrics except Precision, achieving notably high scores of 0.9068 (mIoU) and 0.9479 (mDSC). The removal of any individual module leads to a significant performance drop, highlighting the complementary roles of these components. Furthermore, the fluctuation of the Hausdorff distance (HD) reveals the impact of different modules on the segmentation of boundary details. Our MRANet achieves the best performance on the HD metric (2.8281), indicating that it is more precise in segmenting boundaries. These results above suggest that each module of MRANet plays an important role in improving overall performance.

**TABLE 5:** Ablation study of the components of the MRANet, including PVT, CMKD, RSAB, ASIM, and RSFM.

Module					Performance metrics					
PVT	CMKD	RSAB	ASIM	RSFM	mIoU	mDSC	Recall	Prec.	F2	HD
✓	—	✓	✓	✓	0.8864	0.9308	0.9128	0.9479	0.9239	3.0968
✓	✓	—	✓	✓	0.8891	0.9239	0.9314	0.9379	0.9301	2.9446
✓	✓	✓	—	✓	0.8974	0.9247	0.9403	0.9406	0.9366	3.0107
✓	✓	✓	✓	—	0.8901	0.9346	0.9432	0.9368	0.9316	3.1544
✓	✓	✓	✓	✓	**0.9068**	**0.9479**	**0.9505**	**0.9517**	**0.9490**	**2.8281**

*Note:* ✓indicates the module is enabled; — indicates the module is disabled. The bold values indicate that the corresponding metric achieves the best performance among all comparison methods.

## 5 Discussion

Colorectal cancer (CRC), a common type of cancer, is often diagnosed at later stages because it develops slowly and lacks clear early symptoms. Accurately locating polyps during colonoscopy helps doctors detect and remove them promptly, which is crucial for reducing CRC occurrence and death rates. However, existing segmentation methods struggle with complex polyp shapes, size variations, and generalisation across diverse datasets. To address these issues, we propose the MRANet, which combines dilated convolutions, residual networks, and attention mechanisms to improve the Precision, robustness, and adaptability of polyp segmentation.

First, convolutions with a high dilation rate excel at capturing global contextual information but may overlook local details, while convolutions with a low dilation rate focus on extracting fine‐grained details but struggle to capture the global context. To address this issue, MRANet introduces Convolutions with Multiple Kernels and Dilation rates (CMKD), which combine convolutional kernels with varying dilation rates and kernel sizes to effectively capture both fine details and broader contextual information.

Second, high‐level features typically represent more abstract semantic information from the input data. The proposed Residual Self‐Attention Block (RSAB) leverages a self‐attention mechanism to capture global context, enabling the model to dynamically emphasise critical semantic relationships. This approach enhances the representational power of high‐level features, reduces information loss, and improves the model's ability to express complex tasks with greater robustness. Additionally, RSAB may provide global context for low‐level features, guiding them to emphasise more representative local regions or patterns.

Next, we propose (Attention‐based Scale Interaction Module) ASIM, which employs a feature interaction strategy to effectively integrate detailed information across different scales. The features of various scales are first concatenated and fused, followed by convolutional layers and an attention mechanism to dynamically adjust the feature weights. This approach ensures that important features, including edge and local texture details, are preserved, thus significantly enhancing the Precision of polyp segmentation.

Finally, low‐level features contain more spatial detail, including edges, textures, and local structures. However, complex scale fusion methods may lead to the loss of these detailed features. To resolve this problem, we propose the Residual‐based Scale Fusion Module (RSFM), which merges low‐level features through simple residual connections, effectively preserving spatial details and preventing information loss. Moreover, the residual connections further alleviate gradient issues, thereby enhancing the model's training stability.

In the out‐of‐distribution experiments, MRANet achieves the highest Recall but exhibits relatively lower Precision. This is mainly because the model sometimes misclassifies the background regions as lesion areas, resulting in a higher number of false positives. Therefore, the low Precision of MRANet likely arises from two factors: (1) the CMKD and RSAB modules, despite their strengths, occasionally amplify irrelevant background features; and (2) the diverse and complex nature of lesions in the datasets, such as small sizes, blurred boundaries, and noise interference, which challenge the model's ability to handle unclear lesion regions.

Future efforts will focus on refining feature extraction and fusion strategies, enhancing CMKD and RSAB to improve feature discrimination, and expanding training datasets to boost robustness and generalisation. Additionally, incorporating adaptive mechanisms to balance Recall and Precision will be a key priority to overcome these problems.

## 6 Conclusion

This paper presents a novel Multi‐dimensional Residual Attentional Network (MRANet) for precise polyp segmentation. In the encoding phase, Residual Self‐Attention Block (RSAB) captures high‐level semantic information and guides the refinement of low‐level features by adaptively adjusting attention weights. To further optimise feature extraction, we employ Convolutions with Multiple Kernel and Dilation rates (CMKD) to expand the receptive field, improve encoder representations, and accelerate convergence. In the decoding phase, Attention‐based Scale Interaction Module (ASIM) efficiently fuses upsampled high‐level features with low‐level details, enriching semantic content and capturing fine‐grained features. Additionally, Residual‐based Scale Fusion Module (RSFM) preserves high‐frequency details, such as textures and edges, while leveraging residual connections to support deeper networks, faster training, and robust multiscale feature fusion. Experimental results indicate that MRANet demonstrates the best performance on in‐distribution testing, while also exhibiting strong generalisation capabilities on out‐of‐distribution tests.

## Author Contributions

**Li Zhang:** conceptualization, investigation, methodology, writing – original draft, writing – review and editing. **Yu Zeng:** validation, visualization, writing – original draft. **Yange Sun:** formal analysis, methodology, supervision. **Chengyi Zheng:** conceptualization, formal analysis, software. **Yan Feng:** funding acquisition, methodology, project administration, supervision. **Huaping Guo:** conceptualization, data curation, methodology, supervision.

## Conflicts of Interest

The authors declare no conflicts of interest.

## Data Availability

Our code, dataset (pre‐processed) and experimental results are available at https://github.com/hpguo1982/MRANet.
